# Community health volunteers’ contribution to tuberculosis patients notified to National Tuberculosis program through contact investigation in Kenya

**DOI:** 10.1186/s12889-020-09271-7

**Published:** 2020-07-29

**Authors:** Tabitha Abongo, Benson Ulo, Sarah Karanja

**Affiliations:** Amref Health Africa in Kenya, Global Fund TB project, Nairobi, Kenya

**Keywords:** Contact screening, Tuberculosis, Contact tracing, Community health volunteers, Community health workers

## Abstract

**Background:**

Contact investigation is important in finding contacts of people who have Tuberculosis (TB) disease so that they can be given treatment and stop further transmission. The main objective of this study was to assess the contribution of community health volunteers (CHVs) to the number of TB patients notified to the National program in Kenya through household contact screening and referral of persons with TB signs and symptoms to the facilities for further investigation.

**Methods:**

This was a retrospective desk review of project reports submitted to Amref Health Africa in Kenya by the sub-recipients implementing activities in the 33 counties with Case Notification Rate (CNR) of less 175/100,000 and Treatment Success Rate (TRS) of less than 88% as per the National strategic plan 2015–2018. Data for this study covered a period between January and December 2016. Data on the notified TB patients was obtained from the National Tuberculosis Information Basic Unit (TIBU). The study population included all the TB index cases whose households were visited by CHVs for contact screening. Data was recorded into excel spreadsheets where the descriptive analysis was done, proportions calculated and summarized in a table.

**Results:**

Community health volunteers visited a total of 26,307 TB patients (index cases) in their households for contact screening. A total of 44,617 household members were screened for TB with 43,012 (96.40%) from households of *bacteriologically* confirmed TB patients and 1606 (3.60%) from households of children under 5 years. The proportion of the persons referred to the number screened was 19.6% for those over 5 years and 21.9% from under 5 years with almost the same percentages for males and females at 19.2% and 19.7% respectively. The percentage of (TB) cases identified through tracing of contacts in these counties improved to 10% (5456) of the 54,913 cases notified to the National TB Program.

**Conclusions:**

This study showed that in the 33 counties of Global Fund TB project implementation, the percentage of TB cases identified through tracing of contacts improved from 6 to 10% while the percentage of notified TB cases; all forms contributed through community referrals improved from 4 to 8%. Community health volunteers can play an effective role in household contact screening and referrals for the identification of TB.

## Background

The World Health Organization, Global Tuberculosis report in 2017 identified TB as the leading cause of death from a single infectious agent, ranking above HIV/AIDS. In 2016, there were an estimated 1.3 million TB deaths among HIV-negative people and an additional 374,000 deaths among HIV-positive people. An estimated 10.4 million people fell ill with TB in 2016 [1].

Through its End TB strategy, World Health Organizations encourages regular screening of persons who are in close contact with TB patients for timely Tuberculosis diagnosis [2, 3].

Studies conducted in high burden areas have shown that active case finding among household contacts yields substantially more TB cases than passive case detection [4–6].

Kenya is among the 30 high Tuberculosis burden nations in the world and is among the top 5 from sub-Saharan Africa. In Kenya, Tuberculosis is one of the biggest disease killers [1].

A Tuberculosis study done in Kenya in 2016 reveled that almost 50% of the persons with Tuberculosis are not notified for TB [7].

Tuberculosis (TB) contacts are people who have close contact with patients with infectious TB because they are at high risk for infection (and in line with the End TB strategy), TB contacts should be investigated systematically and actively for TB infection and disease [8].

Contact investigation is important in finding contacts who have Tuberculosis (TB) disease so that they can be given treatment and stop further transmission [6].

The International development organization, Voluntary Service Overseas has highlighted the benefits of community health volunteering, such as creating links between the formal health system and the community, increasing health care access and community empowerment [8]. Community health workers are known by many different names in different countries, but according to WHO, community health workers should be members of the communities where they work, should be selected by the communities, should be answerable to the communities for their activities, should be supported by the health system but not necessarily a part of its organization and have shorter training than professional workers [9].

In Kenya, screening contacts of Tuberculosis patients is mainly done by CHVs. However, in some cases, health care workers with the assistance of facility based CHVs undertake contact initiation through phone calls or patients themselves. Due to stigma, the majority of patients at the time of diagnosis are reluctant to involve their contacts. Additional education by CHV is useful to help bridge the gap.

The main objective of this study was to assess the contribution of community health volunteers (CHVs) to the number of TB patients notified to the National program in Kenya.

The specific objectives were to: 1) establish the total number of households of TB index cases visited by CHVs for contact screening; 2) identify the total number of household members screened for TB; 3) establish the total number of persons referred for further TB investigation; and 4) assess the contribution of CHVs on the total number of TB cases notified to the national TB program

## Methods

Amref Health Africa in Kenya (Amref), a non-state Principal Recipient (PR) for Global Fund Tuberculosis (TB) project, implemented TB activities through 29 Sub- recipients in the year 2016. Global Fund supported Amref in Kenya to implement TB activities in 33 counties with Case Notification Rate (CNR) of less 175/100,000 and Treatment Success Rate (TRS) of less than 88% as per the National strategic plan 2015–2018 [10]. In 2015 the percentage of (TB) cases identified through tracing of contacts in these counties was 6% (3365) of the 59,921 cases notified to the National TB Program while the percentage of notified TB cases, all forms contributed through community referrals was only 4% (2228).

### Study population

The study population included all the TB index cases and children under 5 years whose households were visited by CHVs for contact screening between January and December 2016 and reported to Amref. A total of 2691 Community Health Volunteers (CHVs) in the 33 counties of implementation were taken through 3 days training on community TB care management; some of the topics covered during this training included, introduction to TB basic information, drug resistance, TB/HIV association, Nutrition management of TB, Infection prevention and control of TB. The CHVs were also trained on the community-based reporting tools with an emphasis on data collection and recording from the households, contact screening as well as referral for persons with signs and symptoms of Tuberculosis.

In addition, a total of 2398 Community Health Extension Workers (CHEWs) were trained on Tuberculosis and supervision of community-based activities that included TB control with an intensified focus on supervision of the Community Health volunteers. CHVs were then linked to 2404 health facilities registering at least one TB patient within the 33 counties of intervention. CHEWs generated a list of the bacteriologically confirmed TB patients and children under 5 years with TB and allocated them to the CHVs who visited their households for health education, screening, and referral for cases with TB signs and symptoms.

### Study design and setting

This was a retrospective desk review of project reports submitted to Amref by the sub-recipients implementing activities in the 33 counties with Case Notification Rate (CNR) of less 175/100,000 and Treatment Success Rate (TRS) of less than 88% as per the National strategic plan 2015–2018(Table 1). Data for this study covered a period between January and December 2016. Data on the notified TB patients was obtained from the National Tuberculosis Information Basic Unit (TIBU) a national platform for all Tuberculosis patients’ data.

**Table 1:** The 33 counties of Tuberculosis implementation in Kenya, 2016

1	Baringo	12	Kisumu	23	Nyamira
2	Bungoma	13	Kwale	24	Nyandarua
3	Busia	14	Laikipia	25	Nyeri
4	Homabay	15	Machakos	26	Samburu
5	Isiolo	16	Mandera	27	Siaya
6	Kakamega	17	Meru	28	Taita Taveta
7	Elgeyo Marakwet	18	Murang’a	29	Tana river
8	Kiambu	19	Nairobi	30	Tranzoia
9	Kirinyaga	20	Nakuru	31	Vihiga
10	Kisii	21	Nandi	32	Wajir
11	Kitui	22	Narok	33	West Pokot

Household contact screening was done for all contacts of *bacteriologically* confirmed TB patients and contact of all children under 5 years with TB regardless of the type of TB. Household TB screening for contacts of *bacteriologically* confirmed TB patients and contact of all children under 5 years included evaluation for possible TB disease with a symptoms questionnaire with six key questions that included: Cough of any duration, History of close contact with confirmed TB patient, body fever, noticeable weight loss, chest pain or breathlessness and finally night sweats. In case any of the six questions were answered “yes” then the person was considered presumptive for TB and referred to the health facility for further TB investigation. The clinician at the facility did physical examination of the contacts and symptomatic persons were sent for Gene Xpert test or examination by smear microscopy where there was no Gene Xpert machine.

The CHVs ensured that all children under 5 years who had close contact with the index cases were referred irrespective of the screening outcome for further investigations and possible Isoniazid preventive therapy (IPT) initiation. Community Health Volunteers were provided with transport and lunch allowance of USD 8.4 for every household visited and family members screened. The CHEWs were supported with airtime allowance for effective supervision and coordination of the CHVs activities.

The community health volunteers used the Ministry of Health (MOH) approved screening, referral, and contact tracing forms, which they filled in during household visits. The forms were verified by the CHEWs for completeness and correctness. The CHVs then followed up the referred TB presumptive contacts through household visits or telephone calls and ensured they all arrived at the link health facility (TB clinic) for further investigations.

CHVs together with the Health Care Workers (HCW) at the TB clinic ensured that all contacts were registered in the contacts register and their outcomes updated appropriately. The contacts diagnosed with TB were recorded in the facility TB register and *‘referred by’* column updated appropriately to make sure the yield from the CHVs household contact screening was well captured and reported.

For each index case visited and contacts screened, the contact investigation form was attached to the screening and referral forms. These forms were verified and certified by the sub-county TB coordinators and then submitted to the sub-recipients (SRs) implementing the Global Fund TB project in the specific counties. Original copies of the forms were submitted to Amref the Principal Recipient (PR), second copy remained with the SR and the last copy was filled at the facility. Data from all the verified forms collected by the SR were entered into the Grants Management Information System, a web-based system that works as the project database for all the implementation data.

### Data validation

The Sub-recipient held monthly meetings with the Community Health Volunteers to validate data collected and share any challenges. Also, the Sub-recipients held monthly facility meetings with the TB nurse, facility in-charge, and representatives from the community health volunteers to discuss the work done by the community health volunteers and validate data submitted every month. Amref carried out Quarterly Onsite Data Verification (OSDV) to assess the quality of reported programmatic results. The on-site checks were made to provide valuable information to the project team on where potential issues and gaps could be and allow the project to plan appropriate follow-up actions to address these issues. Random sampling, which is important to minimize selection bias, combined with purposive selection, was used to select the counties, sub-counties, and facilities visited. Using the sampled forms submitted to Amref, the project team through the support of the county and sub-county TB coordinators verified the index cases visited for contact screening by checking their names from the TB register to confirm that they were true TB patients. The team worked with the TB nurses at the facility level to confirm that the households were visited for contact screening, the team also verified duplicate copies of the contact screening and referrals forms filed at the facility level.

### Variables

The primary outcome variable was TB cases notified to the national TB program through referral by community health volunteers. When persons with TB signs and symptoms are referred for investigation by CHVs, they start from the laboratory where they provide sputum that is tested for TB using GeneXpert or TB microscopy. Those diagnosed with TB are enrolled and initiated on treatment. TB coordinators enter the facility data into a central electronic database, TIBU, using mobile computer tablets. Other covariates in this study include TB index cases visited for contact screening, the number of contacts screened, the number referred for further investigation, and TB cases identified from referrals by CHVs. Demographic variables of the subjects such as gender and age were also included.

### Availability of data and materials

Amref Health Africa was competitively selected as the Principal Recipient for Global Fund TB in Kenya for non-state actors with a mandate to implement community-based TB control activities in partnership with the National TB program, Sub-Recipients, and communities living with the disease. The data used in this study was purely from the project reports submitted to Amref by the sub-recipients. Approval to use national data on notified TB patients to be visited by CHVs was provided by the national TB program, the custodian of all TB patients’ data, as part of the grant implementation partnership. For purposes of confidentiality, the data received on cases notified to the National TB Program contributed through community referrals was an aggregate of case finding and referrals from the 33 counties generated from Tuberculosis Information Basic Unit (TIBU) without the patient names.

### Data processing and analysis

Data was obtained from the project monthly reports submitted to Amref by the sub-recipients. The project team verified all contact tracing, screening, and referrals forms for completeness. The team also verified that all the index TB patients visited for contact screening were either *bacteriologically* confirmed or children under 5 years with any type of TB. Any forms with incomplete details were returned for correction by CHVs under supervision and verification by the sub-recipients. Data for TB patients over 5 years who were not bacteriologically confirmed was excluded from this study. Data was recorded into excel spreadsheets where the descriptive analysis was done, proportions calculated and summarized in a table. Simple frequencies and percentages described the number and proportion of household contacts identified, screened and referred.

## Results

Community health volunteers visited households of 26,307 index TB patients comprised of 25,534 (97.1%) *bacteriologically* confirmed and 773 (2.9%) TB patients under 5 years with all forms of TB. Majority of the TB patients visited were female 12,954 (50.7%) compared to 12,580 (49.3%) male (Table 2).

**Table 2:** Bacteriologically confirmed patients and children under five years visited for Tuberculosis contact screening in 33 counties in Kenya, 2016

Indicators and variables	Gender	Bacteriologically confirmed patients		All under 5 years		Total N
		n	%	n	%	
Total Number of index cases visited for contact screening	Male	12,580	49.3%	403	52.13%	
	Female	12,954	50.7%	370	47.87%	
	Total	25,534	100%	773		26,307

Of the 44,617 household members screened for TB, 43,012 (96.4%) were from households of *bacteriologically* confirmed TB patients and 1605 (3.6%) from households of TB patients under 5 years. Majority of the bacteriological confirmed TB patients contacts screened were female, 23,651(53%) compared to and 20,966(47%) male and 904 (56.3%) female compared to 701(43.7%) male contacts for children under 5 years (Table 3). A total of 8679 persons were found to have signs and symptoms of TB and were referred to health facilities for further investigation (Table 4). This number included 8357(96.3%) from households of *bacteriologically* confirmed TB patients and 322(3.7%) from households of TB patients under 5 years.

**Table 3:** Contacts of bacteriologically confirmed patients and children under 5 years screened for Tuberculosis in 33 counties in Kenya, 2016

Indicators and variables	Gender	Bacteriologically confirmed patients		All under 5 years		Total N
Total number of contacts screened for TB	Male	20,265	47.11%	701	43.7%	
	Female	22,747	52.89%	904	56.3%	
	Total	43,012	100%	1605		44,617
	% screened	96.4%		3.60%		100%

**Table 4:** Persons referred for Tuberculosis further investigation in 33 counties in Kenya, 2016

Indicators and variables	Gender	Bacteriologically confirmed patients		All under 5 years		Total N
Total number of persons referred for further investigation	Male	3893	46.58%	124	39%	
	Female	4464	53.42%	198	61%	
	Total	8357	100%	322	100%	8679
Proportion of number referred compared to screened	Male	19.21%		17.69%		
	Female	19.62%		21.90%		

The proportion of screened contact referred was 19.4% for *bacteriologically* confirmed TB patients and 20.1% for TB patients under 5 years with almost the same percentages for male and female at 19.2 and 19.7% respectively (Table 4).

The percentage of (TB) cases identified through tracing of contacts in these counties improved to 10% (5456) of the 54,913 cases notified to the National TB Program (Table 5).

**Table 5:** Community Health Volunteers contribution to TB patients notified in 33 counties in Kenya, 2016

TB patients notified	2015	2016
TB patients notified to TB program from 33 counties	59,921	54,913
TB cases from Community Health Volunteers referrals	2228 (4%)	4489 (8%)
TB cases identified through Contact Invitation	1137 (2%)	977 (2%)
**Totals TB patients notified from contact tracing**	**3365 (6%)**	**5466 (10%)**

## Discussion

This study showed that in the 33 counties of Global Fund TB project implementation the percentage of TB cases identified through tracing of contacts improved from 6 to 10% while the percentage of notified TB cases; all forms contributed through community referrals improved from 4 to 8%. The results clearly show that community health volunteers play an effective role in household contact screening and referrals for identification of TB. These findings are consistent with other studies that have shown that the utilization of community health volunteers for household contact screening can positively affect the total patients notified to the national TB program [11–16].

The increased number of TB cases reported from the community health volunteers’ household screening and referral could be attributed to the training of Community Health Volunteers and the Community Health extension workers to support active case finding activities that included Household TB screening. Better documentation of community referrals in the TB register was also observed during this period. The improved documentation may be as a result of Amref supported quarterly sub-county data review meetings supported though the sub-recipient in collaboration with the TB coordinators to review the quality of TB data reported. In addition, the Amref continuously supported lead CHVs in 16 counties that had a low case notification rate of less than 175/100,000 population. In these counties, the referred contacts with signs and symptoms were guided to the appropriate facility department and enrolled on treatment where necessary. Amref also supported the CHEWs with a monthly communication allowance of USD 2.5 for the coordination and supervision of community health volunteers. The community health volunteers were supported with transport and lunch allowance of USD 8.4 for every household visited for contact screening.

**Fig. 1:**
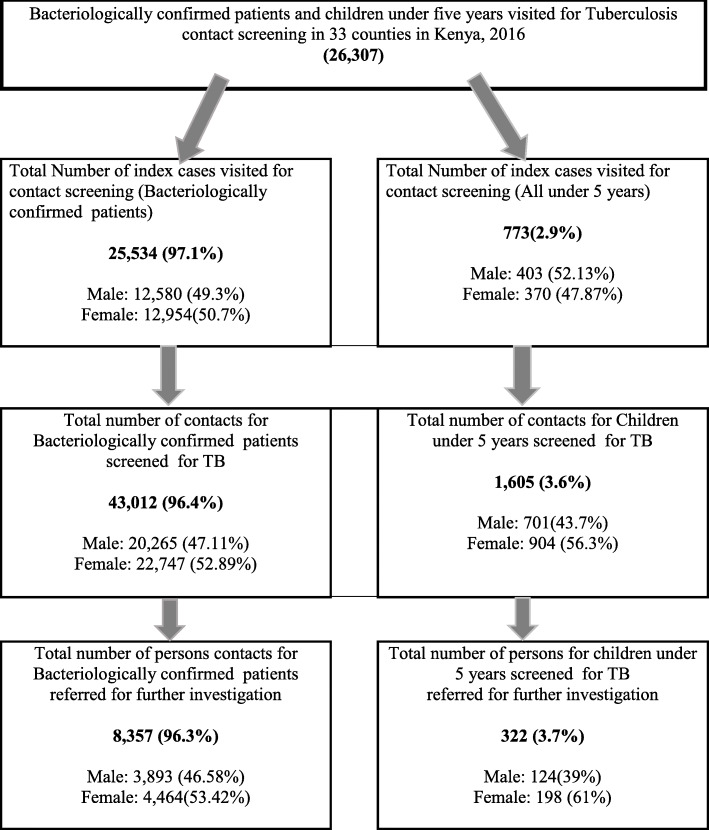
Contact screening flow diagram in 33 counties in Kenya, 2016

The number of TB patients under 5 years whose households were visited for screening were very low (2.91%) compared to those *bacteriologically* confirmed TB patient (97.1%) (Fig. 1). The low number is in line with the Kenya prevalence survey in 2016 that revealed that out of the possible 22,000 paediatric TB cases only 7714 were diagnosed, representing 35% of all cases in 2016. The survey findings showed that the use of TB microscopy for diagnosis that is still common in Kenya misses more than 50% of cases [7].

From the study results, more female contacts (53%) were screened for TB compared to the male contacts (47%) which imply that there were more female at the household level compared to men available for TB screening. The Kenya prevalence survey 2016 results showed that the prevalence of TB was twice as high in men compared to women. The survey identified men between 25 and 34 years to bear the highest burden of TB in Kenya yet this programmatic analysis showed that they are not available for household contact screening. The findings from this study show the need for targeted screening and active case finding among men as a TB high risk group.

The findings on the total cases notified to the national program improved from 3335 (6%) to 5466(10%) during the study period and therefore the programmatic data from this study strongly supports the intervention of utilizing community health volunteers in TB case finding through household contact screening.

The drop in the overall cases notified in 2015 (59,921) compared to 2016(54,913) was due to errors in situation analysis conducted for the *National TB strategic plan 2015–2018* that limited the Global Fund support to only 33 counties. This left out 14 counties that were erroneously classified as having the ability to sustain themselves and therefore did not require intense Global Fund support. The 33 counties of implementation were also low and medium TB burden. After 1 year of implementation, the 14 counties had to be included in Global Fund grant support.

### Study limitations

The study did not have major limitations but there was lack of comparison control group which could have helped in controlling for any factors that may have influenced the relationship between the Community Health Volunteers and the number of TB cases notified. The study did not control for any confounding factors. Also, the proportion of referred clients who reached the health facilities was not closely tracked hence difficulty determining pre-diagnosis losses. The study results gives a clear association between the CHVs but not possible to determine the causation.

## Conclusion

The findings on the total cases notified to the national program improved from 3335 (6%) to 5456(10%) during the study period and therefore using programmatic data by this study strongly supports the intervention of utilizing community health volunteers in TB case finding through household contact screening. The TB prevalence survey showed that there was more TB among men than women. There is need for targeted screening for men since they are missed during household visits. Innovative interventions targeting the informal labour sector as well as schools and colleges could increase the number of males accessed. Investing in CHVs to carry out active contact screening and operationalizing community-based structures in referrals for TB can offer significant gains for TB control in high TB burden countries like Kenya.

## Data Availability

The datasets generated and analyzed during the current study are not publicly available because the data belongs to the Kenya National TB program who is the custodian of all TB patients’ data. Data are however available from the corresponding author (Tabitha Abongo through the email address: tabitha.abongo@gmail.com) upon reasonable request and with permission from national TB program

## Abbreviations

AIDS: Acquired Immunodeficiency Syndrome
CHEWs: Community Health Extension Workers
CHVs: Community Health Volunteers
CNR: Case Notification Rate
HCWs: Health Care Workers
HIV: Human immunodeficiency Virus
IPT: Isoniazid preventive therapy
MOH: Ministry of Health
OSDV: Onsite Data Verification
PR: Principal Recipient
SR: Subrecipient
TB: Tuberculosis
TIBU: National Tuberculosis Information Basic Unit
TSR: Treatment Success Rate
WHO: World Health Organization
